# Flexor Tendon Continuity and Negative X-ray: The “Combo” Negative Features in Finger Subamputation

**DOI:** 10.3390/jcm13113331

**Published:** 2024-06-05

**Authors:** Pierfrancesco Pugliese, Mariangela Vulpetti, Greta Tondini, Francesca Toia

**Affiliations:** Plastic and Reconstructive Surgery, Department of Precision Medicine in Medical, Surgical and Critical Care, University of Palermo, 90127 Palermo, Italy; mariangela.vulpetti@unipa.it (M.V.); greta.tondini@unipa.it (G.T.); francesca.toia@unipa.it (F.T.)

**Keywords:** finger subamputation, avulsion injury, revascularization, regularization

## Abstract

**Background**: The subamputation of fingers with vascular compromise presents a surgical challenge. Although tissue continuity may be considered a favourable prognostic element, in our experience, we noticed that there is not always a direct correlation between soft tissue involvement, radiographic appearance and final outcome. **Methods**: We included, in our study, all cases of vascular pedicle injury in which finger salvage was attempted with microsurgical revascularisation. Exclusion criteria were: integrity of both vascular pedicles, pedicle lesion without global circulatory compromise and patients treated immediately with amputation. **Results**: Between May 2018 and July 2023, 27 male patients with finger subamputation injuries were treated at our institution. In 11 cases of injured fingers, the only intact tissue was the flexor digitorum profundus (FDP) or flexor pollicis longus (FPL). Our global failure rate was 49%; whereas, in the subgroup of the 11 cases with continuity of the FDP or FPL, the failure rate rose to 73% and when the fingers showed flexor tendon integrity and radiographs demonstrated minimal bone damage, revascularisation failure was observed in all cases (100%). **Conclusions**: The results of the study show that subamputations with devascularisation, clinically presented with the combination of flexor tendon as the only element of tissue continuity and dislocation or minimal bone/articular injury, have a worse prognosis because of their trauma mechanism. We propose to add them to the Kay-Adani Classification as a subset of the poorest prognostic injuries group (III), to help surgeons to make decisions about the management of subamputation finger injuries.

## 1. Introduction

Subamputations of the finger with vascular compromise are a surgical challenge. Biemer defined them as injuries with interruption of the main vascular pedicle with circumferentially less than one quarter of a soft tissue connection of the finger, resulting in inefficient circulation. The level of sub-amputation is identified with the focus of the bone fracture, whereas degloving injuries are identified with a wound at the level of the skin [1]. Although the presence of tissue continuity may be considered a favourable prognostic element, there is not always a direct correlation between healthy skin status or the presence of tendon bridges, radiographic imaging and outcome after revascularisation. Among the different aetiologies of trauma, avulsion is the most destructive and difficult to treat injury [2]. In subamputation injuries, the hand surgeon is faced with the need for urgent treatment, with even less time for management than in amputation trauma, due to the inability to preserve the anatomical segment at low temperatures [3]. Therefore, it is important to make a decision about the surgical management as soon as possible when a patient with finger subamputation arrives at a hand trauma centre (so called C.U.M.I.—Coordinamento Urgenze Mano Italia centre in Italy).

Currently, one of the most commonly used classifications for complex finger injuries and their management is the Kay-Adani classification, but it does not emphasise that subamputation with an intact flexor tendon and a mild radiological image (bone dislocation or minimal fracture), although its clinical presentation may at first glance appear favourable, has a negative surgical outcome in reality. In this paper, following a retrospective analysis of our experience in the microsurgical management of subamputation finger injuries, we propose to classify this particular clinical and radiological presentation as a subset of the group of injuries with the poorest prognosis, using the Kay-Adani classification. This can help the surgeon to explain the possibility of failure to the patient and to make a decision not to attempt the revascularisation.

## 2. Materials and Methods

In our retrospective study, we recruited patients who were referred to our hand traumatology centre from our emergency department, from other hospitals of the Sicily and other neighbouring regions and from the activation of the C.U.M.I. emergency line.

The study was approved by the institutional ethics committee and it complied with the Declaration of Helsinki and good clinical practice guidelines.

Informed consent was obtained from all subjects involved in the study.

We included all cases of sub-amputation injury with vascular pedicle damage where finger salvage was attempted with microsurgical revascularisation. Exclusion criteria were the integrity of both pedicles, pedicle lesions without global circulatory compromise and patients treated immediately with amputation.

For each case, we analysed the mechanism of injury and the presence of clinical signs of vascular damage, the type of soft tissue involved (flexor or extensor tendons, vascular pedicles, nerves and skin), bone involvement, the number of revascularisation attempts, the need for vein grafts and the cases requiring only venous sutures. (Table 1)

The analysis was carried out using data extrapolated from the digital register of the plastic surgery unit, collected from the online intranet database and obtained from the X-ray image database of our institute, called Synapse, where each RX image has an anterior-posterior, lateral and oblique projection. Subsequently, control charts and tables were produced.

After revascularisation, patients were closely monitored hourly for the first 12 h, then every 2 h for another 12 h and daily for the duration of hospitalisation (6 + 2 days). Finger skin colour, capillary refill rate and bleeding were assessed. These data were recorded in the medical file.

## 3. Results

Between May 2018 and July 2023, 27 male patients with finger subamputation injuries were treated at our institute. The mean age was 57 years (range 34–81). Left fingers were involved in 15 cases and right fingers in 12 cases. The thumb was involved in four cases, the second finger in 14, the third in two, the fourth in five and the fifth in two. The injured phalanx was P1 in 10 cases, P2 in 12 cases, P1 + P2 in one case and P3 in four cases. Ten of the 27 patients had an avulsion injury, seven had a crush injury and 10 had a cut injury. All were in Kay-Adani class III.

**Figure 1:**
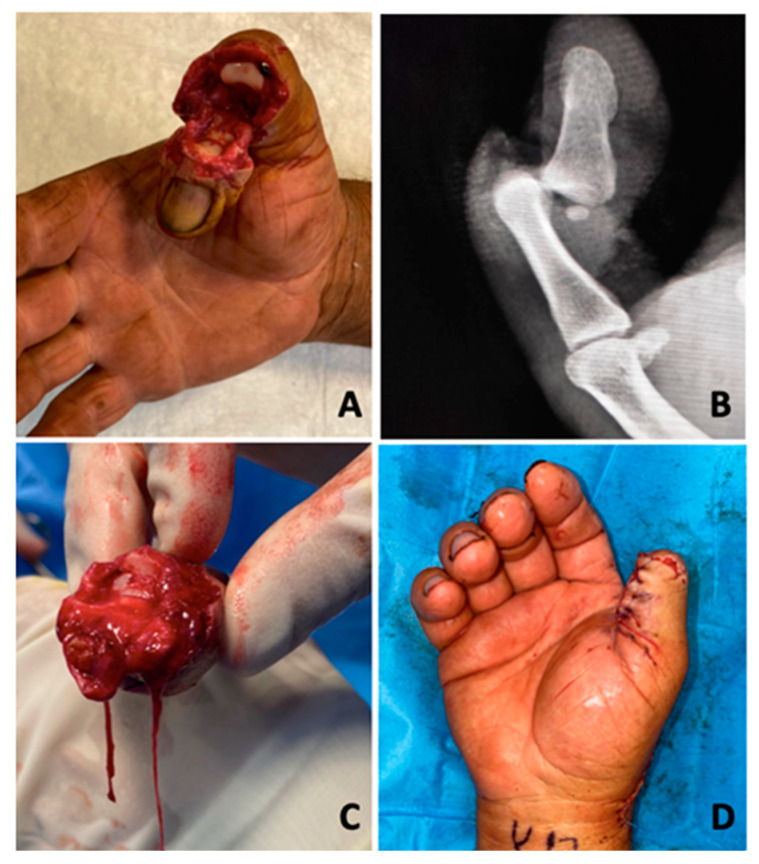
Avulsion injury of the distal phalanx of the thumb. (**A**) Clinical presentation as subamputation, tissue continuity is represented only by the flexor longus pollicis; (**B**) Radiograph shows volar dislocation of the distal phalanx without fracture; (**C**) Clinical image of the extension pedicle after; (**D**) Final aspect after regularisation. Final aspect after reduction.

**Figure 2:**
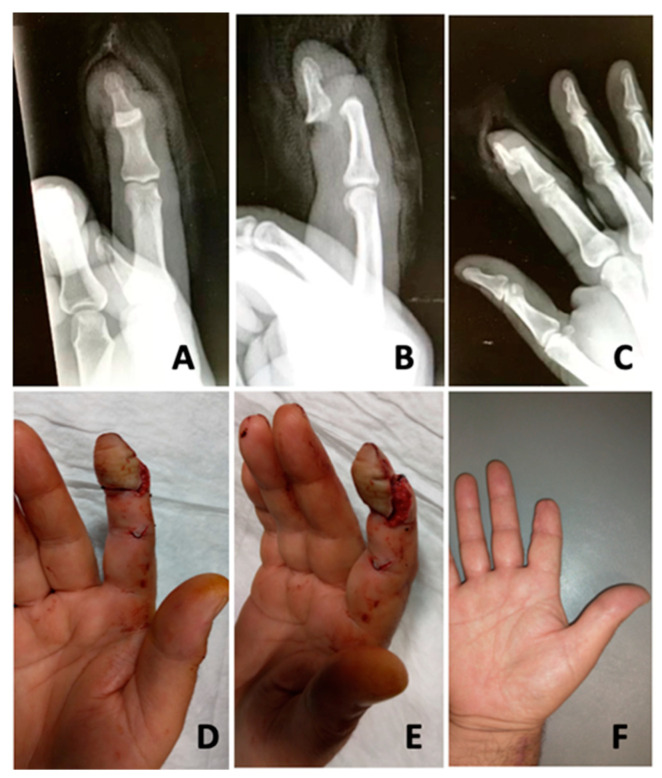
Avulsion injury of the distal phalanx of the second finger. (**A**,**B**) Volar and lateral views of the tissue continuity shown only by the flexor tendon. (**C**–**E**) Frontal, oblique and lateral radiographs showing complete volar dislocation of the distal phalanx without fracture. (**F**) Final aspect after reduction.

Red line and ribbon sign were present in eight cases. In 13 of 27 cases, a vein graft was used to lengthen a short arterial stump. In six out of 27 cases, one or more veins were repaired with an end-to-end anastomosis because of impaired venous drainage. In 11 cases of injured fingers, the only intact tissue was the flexor digitorum profundus (FDP) or flexor pollicis longus (FPL) (Figure 1 and Figure 2).

Considering that in three cases of 27, after primary revascularisation, the finger was regularised after a mean of 8.3 days (range: 2–12) because of secondary necrosis; we reported a global success rate of 51% with adequate revascularization of the finger (14 of 27 cases); global failure rate was 49% (13 of 27 cases).

**Figure 3:**
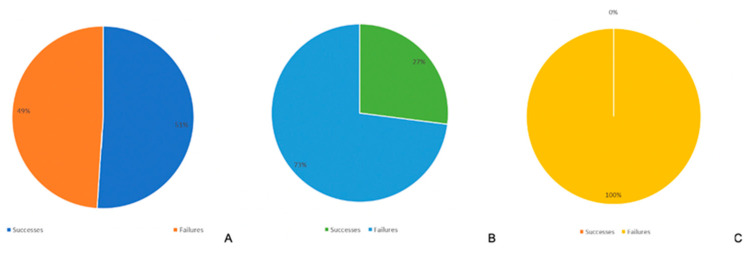
(**A**) Global results. (**B**) Subgroup with continuity maintained only by FP or FPL (11 of 27). (**C**) Subgroup with continuity maintained only by FP or FPL and with dislocation or minimal osteoarticular damage on radiograph (7 of 27).

In the subgroup of the 11 cases with continuity of the FDP or FPL, the subgroup of interest for our study, only three had a successful revascularization, reporting a failure rate of 73%. In addition, when this clinical presentation is combined with a radiographic image proving that there is little bone damage (luxation or fracture not requiring surgical reduction and fixation), we observed failure in all cases (100%) (Figure 3).

## 4. Discussion

Incomplete avulsion injuries have been reported in the literature with varying percentages of successful revascularisation. For this type of trauma, Sears and Chung [4] reported a mean survival rate after surgery of 78%, although the functional outcome measured by TAM (total active motion) is poor, in agreement with other authors [5,6,7,8,9]. These results often lead the surgeon to recommend against attempting surgery.

We analysed the association between the clinical presentation and the final outcome after attempted revascularisation. Our total number of successful revascularisations was 14 out of 27, but in the subgroup of cases showing only continuity of the FLP or FPL, a successful result was achieved in only three patients. In all the cases with a healthy FLP or FPL, with or without a clear description of the accident, we could imagine that the main traumatic mechanism was longitudinal traction. We can assume this because the flexor tendons, due to their elasticity, have a greater resistance to traction and are able to stretch for a few centimetres without bone avulsion. At first glance, these incomplete avulsion traumas, where the distal stump is still attached with a simple circumferential wound, may look like cut-mechanism injuries and typical vascular signs such as ribbon sign and red line are often present. What is more, in some cases, we cannot find an associated fracture and the X-ray can sometimes appear quite normal. Longitudinal traction acts on the less resistant structures such as the joint capsule and ligaments, but it is not a mechanism capable of fracturing a bone, meaning that the cortical tissue resists this type of trauma. Avulsion has been shown to be closely associated with indeterminate widespread injury, making revascularisation more difficult [4], as demonstrated by an experimental study of forearm arteries in monkeys to determine the endpoint of vascular damage after longitudinal traction. Extensive intimal damage with inadequate resection prior to reimplantation is thought to be the main reason for failure. Circumferential skip lesions extend further along the luminal surface of the injured arteries than can be detected by microscopic examination, and histopathological injury is predominantly in the proximal stump. In resected human veins harvested during replantation surgery by the same authors, there were no extensive signs of significant damage [10]. In the literature on the treatment of avulsion injuries, we found different techniques for the management of extensive endothelial damage [2]. Nissenbaum et al. and Weil et al. suggest a posterior dissection of the vessels to achieve a tension-free mobilisation and to perform an end-to-end anastomosis [11,12]. Although we can dissect the vessels proximally to the point of undamaged endothelium, this is not always possible distally, and if the microcirculation is compromised, revascularisation will fail despite a correct anastomosis.

According to Kay [13], vein grafts are required in approximately 70% of cases to fill the gap after excision of the damaged vascular segment. They can be harvested from the volar wrist or a comitant vein of the collateral artery can be used [14]. The rate of successful revascularisation for class III injuries ranges from 73% (Kay) [13] to 100% (Urbaniak) [15]. In our experience, the use of vein grafts in patients with class III injuries does not seem to be crucial for successful revascularisation in special cases, despite long trimming of the injured vessel. Urbaniak described both vein grafts and moving an arterial graft to the opposite side of the digit to have a good distal graft for anastomosis.

In the literature, it is recommended to have at least two patent arteries and three good veins to restore good circulation in finger amputation [15], while other authors suggest that a high rate of finger revascularisation can be achieved with one functioning artery and two functioning veins [3]. We also maintain that it is not always possible to find two collateral arteries, both proximal and distal, but this could be solved by a “Y” shaped vein graft.

The literature describes bone shortening for replantation to reduce tension and remove damaged soft tissue, particularly nerves, vessels and tendons, but this is more feasible for long bones than for the small phalanx. In addition, in the case of a subamputation, where the finger is partially attached, bone shortening is not suitable because of the possible subsequent imbalance of healthy tendons [14].

Nourishment through the vincula [16,17] cannot be considered significant for the vascular supply of the suamputated finger, as the powerful forces of avulsion tend to break these structures, leaving the more resistant flexor tendons intact [18].

In deciding whether or not to attempt revascularisation, it is important to discuss, with the patient, the future time to be invested in treatment and rehabilitation, the risk of lack of good sensation, the poor final functional outcome and the potential risks of secondary procedures [2,14]. In injuries with tissue continuity, the patient usually has higher expectations [3,13], but although we may sometimes have intact venous drainage, on the other hand, the revascularisation outcome is poor due to microvascular damage. In the case of complete avulsion, we can choose not to attempt revascularisation and the patient will easily accept this because of the clinical evidence of the severity of the trauma; in contrast, patients with partial avulsion will hardly accept a negative outcome because the finger is still attached to the hand. So, we are forced to try revascularisation even in these complex situations and, therefore, have a higher failure rate.

In the literature, the classification of avulsion injuries is described by Urbaniak [15], modified by Kay [13] and later by Adani [2], based on the vascular supply, the bone injury and the level of the lesion (proximal or distal to the superficial flexor insertion). This classification system suggests an appropriate treatment for each case and describes the likely outcome.

Class I: circulation adequate, with or without skeletal injury;

Class II: circulation inadequate (arterious IIa or venous IIv-), with no skeletal in jury;

Class III: circulation inadequate (arterious IIIa or venous IIIv-), fracture or articular injury present;

Class IV: complete amputation (IVd distal to FS insertion or IVp proximal to FS insertion [2,13].

The higher the class number, the more complex the injury and surgical treatment [13], and IVp has been shown to have the worst outcome. In the treatment of Kay’s class III injury (which includes all our patients), when osteo-articular and/or tendon injuries are combined, functional recovery will be partial and for these reasons treatment should be carefully evaluated [2]. Therefore, the presence of flexor continuity and radiographic evidence of dislocation or minimal osteo-articular damage not requiring reduction and fixation appeared to be a particularly negative prognostic indicator in our study. As we claim, these situations are intermediate between class III and IV according to the Kay-Adani classification.

However, although our observation seems to follow this trend, statistical significance could not be established due to a reduced sample size.

## 5. Conclusions

The results of the study show that subamputations with devascularisation, where the flexor tendon is clinically the only element of tissue continuity, have a worse prognosis than those where continuity is maintained by other tissues, due to the different mechanism of injury. What makes this trend even more negative is the association between the clinical presentation described above and dislocation or minimal bone/articular injury that does not merit surgical treatment. The main reason for this is to be found in the traumatic mechanism of avulsion, in which vessels, extensor tendon and soft tissues rupture due to longitudinal traction without significant bone damage or flexor tendon disruption. This type of digital trauma with vascular involvement, therefore, presents a mismatch between the clinical radiological presentation and the outcome, and could be separately categorised among the cases with the most unfavourable prognosis.

**Figure 4:**
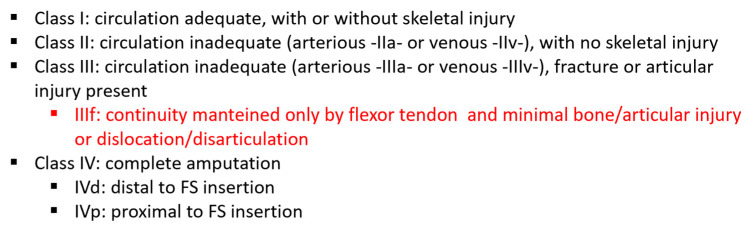
Our proposal to revise the Kay-Adani Classification.

We therefore propose that this type of injury may be included in the class III of Kay- Adani classification (inadequate circulation—arterial and venous, fracture or joint injury present) as a subcategory with a poor prognosis (III-f) (Figure 4).

With this in mind, we believe that an avulsion injury in which the flexor tendon is the only element of tissue continuity, associated with minimal bone/articular injury or dislocation, represents a challenge for the microsurgeon that should be emphasised to the patient because of the high risk of failure. However, although in these particular clinical and radiological presentations, we found a failure of revascularization in whole cases, it is necessary to confirm the results with those of other studies because of the small sample size. At present, it may be important for other hand surgeons to consider our results to pay attention in their cases with these characteristics, to confirm or not to confirm our results, with the future common goal of having a decision algorithm for the management of subamputation finger injuries.

## Figures and Tables

**Table 1:** Patients with finger sub-amputation injuries were treated in our institute.

	Finger	Hand	Flexor t. Injury	Extensor t. Injury	Artery Injury	Nerve injury	Circumferential Skin Lesion	Bone Damage	Phalanx Involved	Mechanism of Injury	Results
1	IV	R	no	yes	yes	yes	yes	joint fx	P2	avulsion	failed
2	II	R	no	no	yes	no	no	fx	P1	crush	vital
3	II	R	no	yes	yes	yes	yes	fx	P2	crush	failed
4	III	L	no	yes	yes	yes	no	fx	P2	crush	vital
5	II	R	no	yes	yes	yes	no	fx	P1	cut	vital
6	II	R	no	no	yes	no	no	fx	P2	crush	failed
7	II	R	no	yes	yes	yes	no	joint fx	P3	avulsion	failed
8	II	R	no	no	yes	yes	yes	dislocation	P3	avulsion	failed
9	II	L	no	yes	yes	yes	no	fx	P2	avulsion	vital
10	IV	R	no	yes	yes	no	yes	dislocation	P3	avulsion	failed
11	I	R	no	yes	yes	yes	yes	dislocation	P2	avulsion	failed
12	IV	L	no	yes	yes	yes	yes	dislocation	P3	avulsion	failed
13	II	L	yes	no	yes	yes	no	fx	P2	cut	vital
14	I	L	no	yes	yes	yes	no	fx	P1	crush	vital
15	V	L	no	no	yes	no	no	fx	P1	avulsion	failed
16	II	L	yes	no	yes	yes	no	fx	P2	avulsion	failed
17	II	R	no	partial	yes	no	no	fx	P1 + P2	crush	vital
18	II	L	yes	yes	yes	yes	no	fx	P1	cut	vital
19	III	L	yes	yes	yes	yes	no	fx	P1	cut	failed
20	II	R	yes	no	yes	yes	no	joint fx	P2	crush	failed
21	II	R	yes	no	yes	yes	no	fx	P1	cut	vital
22	IV	L	partial	yes	yes	yes	no	fx	P2	cut	vital
23	IV	L	yes	no	yes	yes	no	fx	P2	cut	vital
24	I	L	yes	yes	yes	yes	no	fx	P1	cut	vital
25	II	L	yes	yes	yes	yes	yes	fx	P2	cut	vital
26	I	L	yes	partial	yes	yes	no	fx	P1	cut	vital
27	V	L	no	yes	yes	yes	yes	fx + dislocation	P1	avulsion	failed

R: right; L: left; fx: fracture; T: tendon.

## Data Availability

Manuscript data are available on request from the authors.
